# Aggression management of criminal offenders in prison setting

**DOI:** 10.1192/j.eurpsy.2024.342

**Published:** 2024-08-27

**Authors:** G. Nikolić, N. M. Stojanović, J. Branković, S. Tošić Golubović, O. Žikić, J. Kostić

**Affiliations:** ^1^Department of psychiatry; ^2^Department of Physiology, Faculty of Medicine; ^3^Daily hospital, Center for mental health protection; ^4^Clinic for Psychiatry, Faculty of Medicine, Niš, Serbia

## Abstract

**Introduction:**

A new approach of social therapy for criminal offenders was applied in Penalty Facility in Niš, Serbia. It is based on three month peer-training focusing on recognizing of triggers for anger, understanding emotional manifestation and learning socially acceptable ways of anger expression.

**Objectives:**

To estimate how the impact of pear-based training influences the level of agression of criminal offenders in prison settings.

**Methods:**

One hander and six prisoners were randomly assigned to program. The six previously educated inmates trained the participants through 12 work-shops. An independent professional evaluated change in aggression levels after training using Buss&Perry Aggression Scale. We compared subgroups with shorter versus longer sentences pre and post training using Student’s t test. And univariate logistic regression analysis for impacts of sociodemographic variables on aggression scores.

**Results:**

We found a significant higher scores of anger (6.6 ± 4.7 & 11.8 ± 4.2, p=0,043) hostility (15.5 ± 8 & 20.1 ± 6.5, p=0,029) and total aggression (32 ± 14 & 48 ± 21, p=0,023 in subgroup with longer sentences at baseline. After training anger (12.4 ± 4.8 & 15.5 ± 5.6, p= 0,0167), physical aggression (14.6 ± 51. & 17.2 ± 5.6 ,p=0,024) and total aggression score (55.5 ± 14.1 & 68.2 ± 18, p=0,0152) remained higher in the group with sentences more than five years. Lower education level is associated with undesirable outcome-higher level of aggression after training.

**Conclusions:**

Three months training was not sufficient for adopting skills for better control of aggressive behavior in criminal offenders never the less the length of the sentences.

**Disclosure of Interest:**

None Declared

